# Roles of Renal Proximal Tubule Transport in Acid/Base Balance and Blood Pressure Regulation

**DOI:** 10.1155/2014/504808

**Published:** 2014-05-28

**Authors:** Motonobu Nakamura, Ayumi Shirai, Osamu Yamazaki, Nobuhiko Satoh, Masashi Suzuki, Shoko Horita, Hideomi Yamada, George Seki

**Affiliations:** Department of Internal Medicine, Faculty of Medicine, The University of Tokyo, 7-3-1 Hongo, Bunkyo-ku, Tokyo 113-0033, Japan

## Abstract

Sodium-coupled bicarbonate absorption from renal proximal tubules (PTs) plays a pivotal role in the maintenance of systemic acid/base balance. Indeed, mutations in the Na^+^-HCO_3_^−^ cotransporter NBCe1, which mediates a majority of bicarbonate exit from PTs, cause severe proximal renal tubular acidosis associated with ocular and other extrarenal abnormalities. Sodium transport in PTs also plays an important role in the regulation of blood pressure. For example, PT transport stimulation by insulin may be involved in the pathogenesis of hypertension associated with insulin resistance. Type 1 angiotensin (Ang) II receptors in PT are critical for blood pressure homeostasis. Paradoxically, the effects of Ang II on PT transport are known to be biphasic. Unlike in other species, however, Ang II is recently shown to dose-dependently stimulate human PT transport via nitric oxide/cGMP/ERK pathway, which may represent a novel therapeutic target in human hypertension. In this paper, we will review the physiological and pathophysiological roles of PT transport.

## 1. Introduction

Renal proximal tubules (PTs) reabsorb approximately 80% of the filtered bicarbonate from glomerulus, thereby playing a pivotal role in the maintenance of systemic acid-base balance [1]. This process is mostly dependent on Na^+^, which is composed of the luminal Na^+^/H^+^ exchanger and the basolateral Na^+^-HCO_3_^−^ cotransporter [1]. Although distal nephron segments are also involved in the systemic acid/base regulation, acid-base transporters in these segments often cannot completely compensate for defects in bicarbonate absorption from PTs. Indeed, mutations in the Na^+^-HCO_3_^−^ cotransporter NBCe1, which mediates a majority of bicarbonate exit from the basolateral membrane of PTs, are known to cause a severe type of proximal renal tubular acidosis associated with ocular and other extrarenal manifestations [2].

On the other hand, PTs reabsorb approximately 65% of the filtered NaCl, thereby also contributing to the regulation of plasma volume and blood pressure. For example, hypertension is frequently associated with metabolic syndrome, and insulin-mediated stimulation of PT transport may play a role in this association [3, 4]. In addition, angiotensin (Ang) II is pivotal in the regulation of blood pressure, and stimulation of PT transport may play a critical role in Ang II-mediated hypertension [5, 6]. In this review, we will focus on the roles of PT transport in the maintenance of acid-base homeostasis as well as the regulation of blood pressure.

## 2. Roles of PT Transport in Acid/Base Balance

In PTs, the luminal Na^+^/H^+^ exchanger type 3 (NHE3) together with the basolateral Na^+^-HCO_3_^−^ cotransporter NBCe1 is thought to mediate a majority of sodium-coupled bicarbonate absorption from this segment [1, 7]. Although the basolateral membrane of PTs contains Na^+^-dependent and Na^+^-independent Cl^−^/HCO_3_^−^ exchangers [7], these transporters cannot effectively compensate for the loss of NBCe1 function. By contrast, the loss of NHE3 function may be at least partially compensated by the other luminal transporters such as NHE8 [8].

In 1983 Boron and Boulpaep identified the functional existence of electrogenic Na^+^-coupled HCO_3_^−^ transport activity in the basolateral membrane of isolated salamander PTs [9]. Subsequently, Kondo and Frömter revealed that this electrogenic Na^+^-HCO_3_^−^ cotransport activity is robust in S1 and S2 segments but almost absent in S3 segment of isolated rabbit PTs [10]. Yoshitomi and colleagues initially reported that the Na^+^-HCO_3_^−^ cotransporter in rat PTs in vivo functions with 1Na^+^ to 3HCO_3_^−^ stoichiometry [11]. On the other hand, Seki and colleagues revealed that the Na^+^-HCO_3_^−^ cotransporter in isolated rabbit PTs functions with 1Na^+^ to 2HCO_3_^−^ stoichiometry [12]. Later, Müller-Berger and colleagues found that the Na^+^-HCO_3_^−^ cotransporter in isolated rabbit PTs can change its transport stoichiometry depending on the incubation conditions [13]. Interestingly, NBCe1 expressed in* Xenopus* oocytes can also change its transport stoichiometry depending on changes in cytosolic Ca^2+^ concentrations [14]. Consistent with these data, Gross and colleagues reported that stoichiometry of NBCe1 is cell-type specific [15].

In 1997 Romero and colleagues succeeded in the first molecular cloning of NBCe1 from salamander kidney [16]. Among the three major variants, NBCe1A is transcribed from the alternative promoter in exon 1 and abundantly expressed in the basolateral membrane of PTs, representing the major bicarbonate exit pathway in this nephron segment [1]. Another variant NBCe1B is transcribed from the dominant promoter in exon 1 and differs from NBCe1A only at the N-terminus [17]. NBCe1B is first cloned from pancreas but is now known to be expressed in a variety of tissues such as intestinal tracts, ocular tissues, and brain [18–20]. On the other hand, NBCe1C is predominantly expressed in brain and differs from NBCe1B only at the C-terminus [21]. Consistent with the indispensable role of NBCe1 in acid/base homeostasis, Igarashi and colleagues found that inactivating mutations in NBCe1 cause a severe type of proximal renal tubular acidosis (pRTA) associated with ocular abnormalities [2]. Until now 12 different homozygous mutations have been found in pRTA patients [22]. These patients invariably presented with ocular abnormalities such as band keratopathy, cataract, and glaucoma, suggesting that NBCe1 function is essential for the maintenance of homeostasis in ocular tissues. Indeed, NBCe1 is found to be abundantly expressed in several human ocular tissues such as corneal endothelium, lens epithelium, and trabecular meshwork cells [20].

NBCe1 in brain may also play several physiological roles [23]. Indeed, Suzuki and colleagues revealed that defective membrane expression of NBCe1B may cause migraine with or without hemiplegia [24]. NBCe1B activity in astrocytes may be indispensable for the regulation of synaptic pH and neuron excitability.

Two types of NBCe1-deficient mice, NBCe1-KO mice [25] and W516X-knockin mice [26], present with very severe acidemia due to pRTA and die within 30 days. Functional analysis using isolated PTs from W516X-knockin mice confirmed that the normal NBCe1 activity is essential for bicarbonate absorption from this nephron segment [26]. Alkali therapy significantly prolonged the survival of W516X-knockin mice. Detailed analysis of ocular tissues in these mice revealed that NBCe1 plays a critical role in the maintenance of corneal transparency also in mice [26].

Unlike NBCe1-deficient mice, NHE3-KO mice present with only mild acidemia [27]. Although NHE8 seems to partially compensate for the loss of NHE3 function, NHE3/NHE3-double KO mice also present with relatively mild acidemia [8]. So far, mutations in NHE3 or NHE8 have not been found in human pRTA patients.

## 3. Roles of Hyperinsulinemia in Hypertension Associated with Metabolic Syndrome

Certain risk factors such as abdominal adiposity, glucose intolerance, dyslipidemia, and hypertension tend to cluster within individuals. Insulin resistance with obesity is thought to be a key factor for this association, which is now termed as metabolic syndrome [28]. Several different mechanisms such as activation of renin-angiotensin-aldosterone system (RAAS), enhancement of sympathetic nervous system, or hyperinsulinemia may be involved in the occurrence of hypertension associated with insulin resistance [29, 30]. Among these factors, hyperinsulinemia-induced hypertension seems to be an attractive hypothesis in view of the antinatriuretic action of insulin [3, 4]. Indeed, insulin is known to stimulate sodium absorption from several nephron segments. For example, insulin may stimulate sodium absorption from distal convoluted tubules by phosphorylating the Na^+^-Cl^−^ cotransporter NCC through the with-no-lysine kinase 4 (WNK4)/STE20/SPS1-related proline-alanine-rich kinase (SPAK) pathway [31]. In cortical collecting duct (CCD) cells insulin is thought to stimulate sodium absorption by activating the activity of epithelial Na^+^ channel ENaC [32–34], though a recent study failed to confirm the stimulatory effect of insulin on the ENaC activity in isolated mammalian CCD [35]. In PTs, insulin enhances sodium absorption by stimulating the luminal NHE3, the basolateral Na^+^/K^+^-ATPase, and the basolateral NBCe1 [36–39].

Insulin can relax vascular tones through the phosphatidylinositol 3 kinase (PI3K)/Akt-dependent nitric oxide (NO) production, and simple hyperinsulinemia may not necessarily induce hypertension [40]. Notably, however, the vasodilator action of insulin is reported to be attenuated in insulin resistance [41, 42]. Therefore, hyperinsulinemia can be an important factor in hypertension associated with metabolic syndrome, if the stimulatory effects of insulin on renal sodium absorption are preserved even in the systemic insulin resistance.

In support of this hypothesis, recent studies have clarified that defects in insulin signaling at the level of insulin receptor substrate (IRS) proteins are frequently associated with human insulin resistant states, resulting in the occurrence of cell-type specific insulin resistance [43, 44]. The two major substrates IRS1 and IRS2 may mediate distinct pathways in insulin signaling, and they are not functionally interchangeable in many insulin-sensitive tissues [43–45]. Importantly, in adipocytes of human subjects with noninsulin-dependent diabetes mellitus the expression of IRS1 protein is found to be markedly reduced, accompanied with the severe reduction of insulin-mediated glucose uptake [46]. By contrast, the reduction of IRS2 expression seems to be a key factor in several forms of insulin resistance in liver [47].

To clarify the relative importance of IRS1 and IRS2 in the stimulatory effect of insulin on PT transport, Zheng and colleagues compared the effects of insulin on sodium-coupled bicarbonate absorption in isolated PTs from IRS1-KO and IRS2-KO mice. They found that the PI3 K-dependent stimulatory effect of insulin on PT transport was preserved in IRS1-KO mice but markedly attenuated in IRS2-KO mice. Furthermore, insulin-induced Akt phosphorylation was also preserved in IRS1-KO mice but not in IRS-2 KO mice [48]. These results indicate that IRS2 is the main substrate that mediates the stimulatory effects of insulin on PT transport. Importantly, insulin can induce antinatriuresis even in insulin resistant rats and humans [49, 50]. Moreover, PT sodium transport seems to be enhanced in insulin resistant humans [51, 52], suggesting that the stimulatory effect of insulin on PT transport may be preserved in common forms of insulin resistance. Consistent with this view, a recent study showed that the expression of IRS2 as well as insulin-mediated Akt phosphorylation in renal tubules is preserved in Zucker fatty rats that show marked insulin resistance due to defective leptin signaling [53]. In liver, hyperinsulinemia is known to suppress the expression of IRS2, thereby attenuating the insulin signaling in liver [47, 54]. Future studies are required to determine whether the IRS2-dependent stimulatory insulin signaling in PTs is preserved in common forms of insulin resistance.

Interestingly, the IRS1-dependent insulin signaling in glomeruli seems to be attenuated in insulin resistance [53]. Because insulin signaling may be required not only for the nitric oxide (NO) production by glomerular endothelium but also for the preservation of normal podocyte functions [53, 55], insulin resistance in glomeruli may promote the occurrence and progression of diabetic nephropathy. In fact, the treatment with insulin sensitizers thiazolidinediones (TZDs) can protect podocyte from injury independently of glycemic control [56]. However, TZDs, especially when used with insulin, may induce edema formation as a side effect, probably by stimulating sodium absorption from PT and/or distal tubules [57, 58]. Unfortunately, this side effect may offset the beneficial effects of TZDs on the insulin signaling in glomeruli.

## 4. Effects of Ang II on PT Transport

There are two major Ang II receptors (AT), AT_1_ and AT_2_. AT_1_ receptors are further subdivided into AT_1A_ and AT_1B_ in rodents [59]. While AT_1_ may be the main receptors that mediate the effects of Ang II on blood pressure, AT_2_ may be also partially involved in blood pressure regulation [60]. Ang II can regulate blood pressure via AT_1_ receptors in both renal and extrarenal tissues. To clarify the relative importance of these receptors in blood pressure homeostasis, Coffman and colleagues performed kidney cross-transplantation between wild-type and AT_1A_-KO mice [5, 6]. They found that renal and extrarenal AT_1A_ receptors almost equally contribute to the maintenance of baseline blood pressure. However, renal AT_1A_ receptors are indispensable for the occurrence of Ang II-induced hypertension and cardiac hypertrophy. They further showed that specific deletion of AT_1A_ receptors from PTs alone is sufficient to lower blood pressure and provides substantial protection against Ang II-induced hypertension [61]. These results indicate that the stimulatory effect of Ang II on PT sodium transport is quite important in blood pressure regulation.

Paradoxically, however, the effects of Ang II on PT transport are biphasic: transport is stimulated by picomolar to nanomolar concentrations of Ang II, while it is inhibited by nanomolar to micromolar concentrations of Ang II [62, 63]. The effects of Ang II on NHE3, Na^+^/K^+^ ATPase, and NBCe1 in PTs are all known to be biphasic [64–67]. Notably, intrarenal concentrations of Ang II are much higher than those in plasma [68]. Accordingly, the inhibitory effect of Ang II on PT transport could have some physiological significance.

Controversial data have been reported as to the receptor subtype(s) responsible for the biphasic effects of Ang II on PT transport [69, 70]. However, Horita and colleagues, by analyzing the NBCe1 activity in isolated PTs, found that the biphasic effects of Ang II added to bath perfusate were lost in AT_1A_-KO mice [71]. Instead, very high concentrations of Ang II added to bath perfusate induced a slight stimulation of NBCe1 activity, which was probably mediated by AT_1B_ [71, 72]. Zheng and colleagues, by analyzing the bicarbonate absorption rates from isolated PTs, also found that the biphasic effects of Ang II added to luminal perfusate were lost in AT_1A_-KO mice [73]. These results clearly indicate that both luminal and basolateral AT_1A_ receptors mediate the biphasic effects of Ang II on PT transport.

Regarding the signaling pathways, the activation of PKC and/or the decrease in intracellular cAMP concentrations, which may ultimately result in ERK activation, are thought to mediate the stimulatory effect of Ang II [67, 72, 74]. On the other hand, the activation of phospholipase A_2_ (PLA_2_)/arachidonic acid/5,6-epoxyeicosatrienoic acid (EET) pathway and/or the NO/cGMP pathway is thought to mediate the inhibitory effect of Ang II [67, 72, 75]. Consistent with this view, Li and colleagues found that the biphasic effects of Ang II were lost and all the concentrations of Ang II induced a similar stimulation of NBCe1 activity in isolated PTs from cytosolic PLA_2_-KO mice [72].

**Figure 1:**
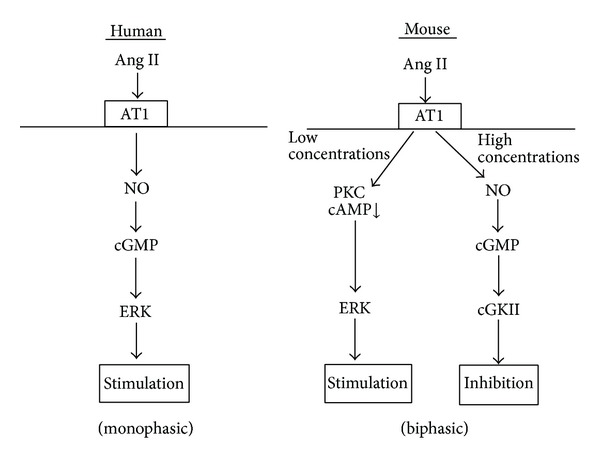
Ang II signaling in mouse and human PTs. In mouse PTs, low concentrations of Ang II induce transport stimulation via either PKC activation or decrease in intracellular cAMP resulting in ERK activation, while high concentrations of Ang II induce transport inhibition via NO/cGMP/cGKII pathway. In human PTs, by contrast, Ang II induces dose-dependent transport stimulation via NO/cGMP/ERK pathway.

While the biphasic effects of Ang II on PT transport have been reported in rats, mice, and rabbits [62, 63, 65, 66, 71, 73], little has been known about the effects of Ang II on human PT transport. To clarify this issue, Shirai and colleagues recently examined the effects of Ang II in isolated human PTs obtained from nephrectomy surgery for renal carcinoma [76]. Surprisingly, they found that Ang II, unlike that in the other species, induced a dose-dependent, profound stimulation of human PT transport via AT_1_-dependent ERK activation. In wild-type mice, the inhibitory effect of Ang II was dependent on the NO/cGMP/cGMP-dependent kinase II (cGKII) pathway. In cGKII-KO mice, the inhibitory effect of Ang II was lost but the NO/cGMP pathway failed to induce the ERK-dependent NBCe1 activation. By sharp contrast, in human PTs, the NO/cGMP pathway mediated the stimulatory effect of Ang II via cGKII-independent ERK activation. Thus, as shown in Figure 1, the contrasting responses to NO/cGMP pathway seem to be largely responsible for the different modes of PT transport regulation by Ang II in humans and the other species.

At present the molecular mechanisms underlying the species differences in PT response to NO/cGMP pathway remain unknown. However, previous studies suggest that such species differences may indeed exist. For example, NO is generally thought to work as inhibitory on PT transport in rodents [77, 78]. Furthermore, salt loading into rodents is known to enhance renal NO synthesis, which may facilitate sodium excretion and preservation of normal blood pressure [79, 80]. In human subjects, however, salt loading fails to induce an adaptive increase in renal NO synthesis [81, 82]. Thus, the role of NO/cGMP in adaptive natriuretic response to salt loading is clearly established in rodents but not in human subjects. Taken together with these considerations, the study by Shirai and colleagues [76] suggests that the unopposed, marked stimulation of PT transport by high intrarenal concentrations of Ang II may play an important role in the pathogenesis of human hypertension. Furthermore, the human-specific stimulatory effect of NO/cGMP pathway on PT transport may represent a novel therapeutic target in hypertension.

## 5. Conclusion

In this paper, we reviewed the physiological and pathophysiological roles of PT transport. Sodium-coupled bicarbonate absorption from PTs plays a critical role in the systemic acid/base balance. Indeed, inactivating mutations in NBCe1 cause severe pRTA associated with ocular and other extrarenal abnormalities. Sodium transport in PTs may also play an important role in blood pressure regulation. In particular, the stimulatory effect of insulin on PT transport may be involved in the pathogenesis of hypertension associated with metabolic syndrome. Unlike in other species, Ang II dose-dependently stimulates human PT transport via NO/cGMP/ERK pathway, which may represent a novel therapeutic target in human hypertension.
